# Study of the Biological Activity of Novel Synthetic Compounds with Antiviral Properties against Human Rhinoviruses

**DOI:** 10.3390/molecules16053479

**Published:** 2011-04-26

**Authors:** Samuela Laconi, Maria A. Madeddu, Raffaello Pompei

**Affiliations:** Sezione di Microbiologia Applicata, Università di Cagliari, via Porcell 4, 09124 Cagliari, Italy

**Keywords:** antivirals, rhinoviruses, pirodavir

## Abstract

Picornaviridae represent a very large family of small RNA viruses, some of which are the cause of important human and animal diseases. Since no specific therapy against any of these viruses currently exists, palliative symptomatic treatments are employed. The early steps of the picornavirus replicative cycle seem to be privileged targets for some antiviral compounds like disoxaril and pirodavir. Pirodavir’s main weakness is its cytotoxicity on cell cultures at relatively low doses. In this work some original synthetic compounds were tested, in order to find less toxic compounds with an improved protection index (PI) on infected cells. Using an amino group to substitute the oxygen atom in the central chain, such as that in the control molecule pirodavir, resulted in decreased activity against Rhinoviruses and Polioviruses. The presence of an -ethoxy-propoxy- group in the central chain (as in compound I-6602) resulted in decreased cell toxicity and in improved anti-Rhinovirus activity. This compound actually showed a PI >700 on HRV14, while pirodavir had a PI of 250. These results demonstrate that modification of pirodavir’s central hydrocarbon chain can lead to the production of novel derivatives with low cytotoxicity and improved PI against some strains of Rhinoviruses.

## 1. Introduction

The viruses in the Picornaviridae family cause an extraordinarily wide range of illnesses [1,2,3,4]. The syndromes associated with these agents include asymptomatic infection, aseptic meningitis syndrome (the most common acute viral disease of the CNS), colds, febrile illness with rash, conjunctivitis, herpangina, muscle infection, heart infection, and hepatitis. Aseptic meningitis is most common in very young infants. Myocarditis and pleurodynia are most prevalent in adolescents and young adults.

Two major human genera of Picornaviridae, the Enteroviruses and Rhinoviruses, have an identical morphology but can be distinguished through clinical, biophysical, and epidemiological studies. The picornavirus viral capsid consists of a densely packed icosahedral arrangement of 60 protomers [5]. Each protomer consists of four polypeptides – VP 1, 2, 3, and 4 – which all derive from the cleavage of a larger protein. The capsid-coat protein serves multiple functions, including: (a) protecting the viral RNA from degradation by environmental RNAse, (b) selecting and packaging viral RNA, (c) penetrating target cells and delivering the viral RNA into the cell cytoplasm, and (d) determining host and tissue tropism by recognition of cell-specific cell-membrane receptors. The virus uses these membrane receptors to enter the target cells [6]. For example, most human Rhinovirus strains bind to the intracellular adhesion molecule 1 (ICAM-1), an immunoglobulin-like molecule, even though some use a low-density lipoprotein receptor [5]. Some of the picornavirus families may use the same receptor which could be shared with unrelated viruses [7,8].

Specific antiviral agents for the treatment of picornavirus infections are not available [9,10,11,12]. Due to the lack of specific antiviral therapy, most enteroviral illnesses are managed symptomatically. Although effective antiviral therapy is not yet clinically available, some promising anti-picornavirus drugs reduce the duration of illness among adults with enteroviral aseptic meningitis [3,4,9,13].

Disoxaril was the first capsid-blocker compound to enter a phase-1 clinical trial; it was shown to have moderate activity against the Rhinoviruses and strong activity against the Enteroviruses, both *in vitro* and *in vivo*; however, additional clinical studies demonstrated high *in vivo* toxicity and the compound did not undergo further development. [14,15,16]. Pleconaril is a new-generation capsid-blocker with a broad spectrum against several Enteroviruses and Rhinoviruses, but the presence of some important side-effects have blocked its clinical use [13,17]. Other “capsid-binding” compounds are pirodavir and its derivatives, which have proved to be effective in intranasal treatment of patients with a common cold caused by some strains of human Rhinoviruses [18]. 

In this study we tested several novel anti-rhinovirus compounds and demonstrated that some modifications of the central hydrocarbon chain of the molecules could lead to new interesting drugs with an improved activity for certain Rhinovirus strains and a decreased cytotoxicity on cell cultures.

## 2. Results and Discussion

### 2.1. Anti-rhinovirus activity 

**Figure 1:**
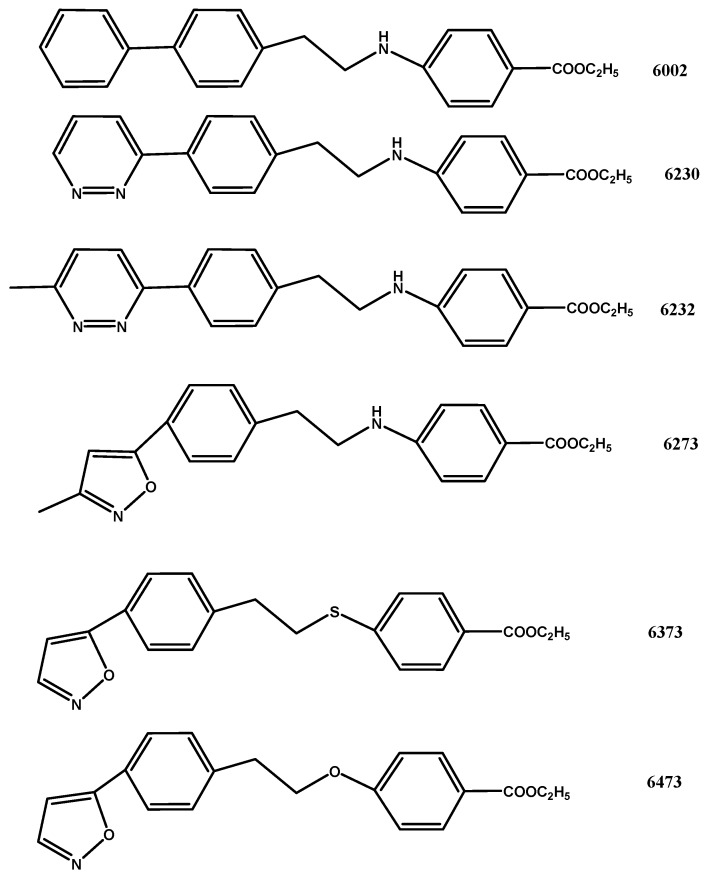

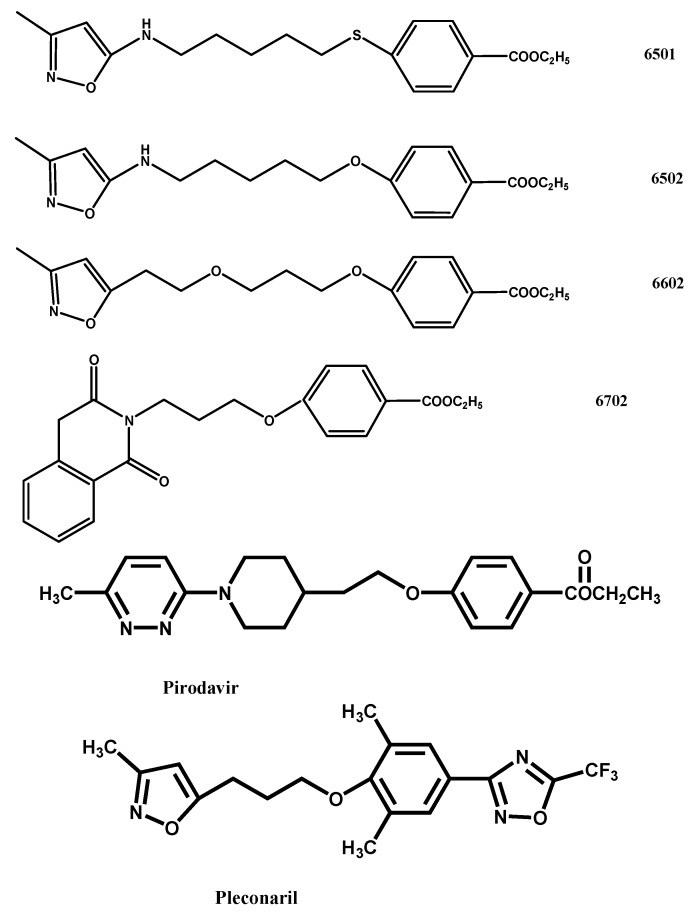
Molecular structures of pirodavir, pleconaril and the novel synthetic compounds.

The molecular formulae of the tested compounds are indicated in Figure 1. Compounds I-6002, I-6230, I-6232 showed poor activity on Rhinoviruses (Table 1), with a protection index (PI) from 1 to 4 on HRV14, although substance I-6232 resulted as being far less toxic than pirodavir (50.0 μg/mL against 3.1, respectively). Also I-6273, I-6373 and I-6473 showed limited antiviral activity against HRV14, with a PI varying from 4 to 16, and from 8 to 33 on HRV39. However, the modifications in the I-6273 molecule, with the insertion of a methylisoxazol group, resulted in decreased cell toxicity on HeLa cells (50.0 μg/mL) as compared to pirodavir. Substance I-6501 showed a PI of 4 on HRV14 and of 40 on HRV39, whereas compounds I-6502, I-6602 and I-6702 were the most active anti-Rhinovirus molecules tested in this study; I-6502 scored a PI of 83 on HRV14 and of 178 on HRV39; I-6602 showed an effective dose of 0.07 μg/mL on HRV14 and HRV39, with a PI of 714 on both virus strains. The last compound tested (I-6702) displayed low toxicity with a cytotoxic dose (CD) of 50 μg/mL and a PI of 714 on HRV14 and of 143 on HRV39; however due to its poor stability, these results were not always repeatable, probably because this compound was inactivated by cellular enzymes. The active anti-Rhinovirus concentrations of pirodavir were 0.012 μg/mL on HRV14 and 0.006 on HRV39, respectively. Although the specific activity of pirodavir resulted as being greater than that of the novel derivatives, the PI of compound I-6602 (= 714) was found to be more than twofold that of pirodavir (= 250), since it was less toxic to HeLa cell cultures (50.0 against 3.1 μg/mL). These compounds all showed poor activity on Polio 1, with inhibiting doses (ID) from 1.99 to 2.65 µg/mL.

Therefore the addition of an -ethoxy-propoxy- group in the central chain, such as in I-6602, produced a compound with low toxicity on cell cultures, but endowed with strong anti-Rhinovirus activity. Although the specific pirodavir activity was still superior to the I-6602 compounds, its toxicity, one of the main causes of pirodavir failure in clinical use, resulted in a much lower PI. 

The derivatives containing a -thioether- moiety in the central chain (6373,6501) showed poor activity against Rhinoviruses, suggesting that the large size of this atom, as compared to that of oxygen, may perhaps lead to difficulty in compound interaction with the VP1-viral canyon adhesin [7]. Moreover, the substitution of the oxygen atom in the central chain with a secondary amino moiety (6002, 6230, 6232, 6273) resulted in a general decrease in the antiviral activity of the tested compounds on Rhinoviruses. In addition, one of the novel tested compounds (I-6232) showed better inhibitory activity than pirodavir on Polio 1 (Table 1). It was also observed that modification in the left moiety of the pirodavir molecule (Figure 1) with the substitution of the methyl-pyridazinyl-piperidinyl group with other chemical entities, such as in I-6232 and I-6602, could lead to the production of compounds whose cytotoxic activity was 10–15 times lower than pirodavir’s.

Two important findings were obtained from this study: firstly, some original derivatives, less toxic on cell cultures than pirodavir, were designed and tested leading to highly improved protection indexes on Rhinovirus infected cells as compared to the control molecule; secondly, the modification of the central chain of the pirodavir molecule, with the substitution of the –oxy- group or the addition of a second oxy or amino moiety, allowed the production of several molecules with extremely variable activity on the different strains of Rhinoviruses tested; some were almost ineffective, while others were very active against HRV14 and HRV39, with inhibiting properties sometimes much stronger than pirodavir’s. 

### 2.2. Time-addiction effect of some novel compounds on HRV14 

**Figure 2:**
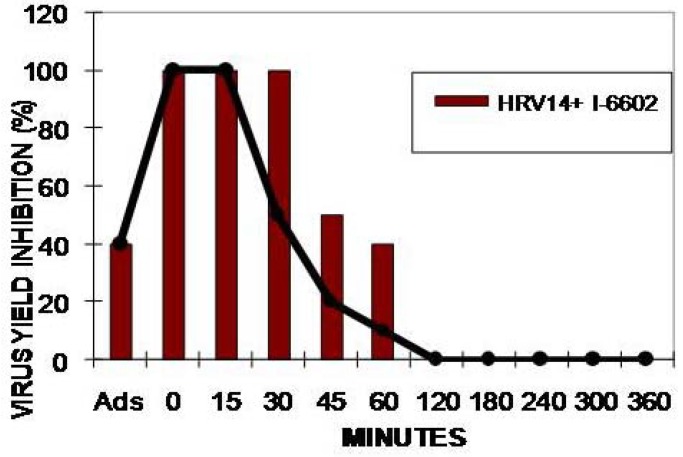
Time-addiction effect of compounds I-6602 (0.5 µg/mL) and pirodavir (0.5 µg/mL) on HRV14 at various times of incubation during the viral replicative cycle.

In the time-addiction assay the I-6602 compound only exerted its anti-Rhinovirus activity in the first hour after infection, similar to pirodavir, with an inhibition peak on HRV14 within about 45 min (Figure 2).

The time addiction tests revealed that most of the novel compounds behaved like pirodavir and only inhibited Rhinovirus growth and multiplication in the early phases of viral reproduction. This confirms the established finding that pirodavir along with its derivatives could interfere with virion adhesion to the cell receptors (e.g. ICAM-1) [7,19].

## 3. Experimental

### 3.1. Compounds

The compounds tested in this work were designed and synthesized by IFI SpA (Rome) and were indicated with the following numbers:

I-6002, (ethyl 4-(2-(biphenyl-4-yl)ethylamino)benzoate),
I-6230 (ethyl 4-(4-(pyridazin-3yl)phenethylamino)benzoate),
I-6232 (ethyl 4-(4-(6-methylpyridazin-3yl)phenethylamino)benzoate),
I-6273 (ethyl 4-(4-(methylisoxazol-5-yl)phenethylamino)benzoate),
I-6373 (ethyl 4-(4-(3-methylisoxazol-5-yl)phenethylthio)benzoate),
I-6473 (ethyl 4-(4-(3-methylisoxazol-5-yl)phenethoxy)benzoate),
I-6501 (ethyl 4-(5-(3-methylisoxazol-5-ylamino)pentylthio)benzoate),
I-6502 (ethyl 4-(5-(3-methylisoxazol-5-ylamino)pentyloxy)benzoate),
I-6602 (ethyl 4-(3-(2-(3-methylisoxazol-5-yl)ethoxy)propoxy)benzoate),
I-6702 (ethyl 4-(3-(1,3-dioxo-3,4-dihydroisoquinolin-2(1H)-yl)propoxy)benzoate).

All these compounds were designed using the basic structure of both pirodavir and pleconaril as a model (see Figure 1), with substantial modifications in the central hydrocarbon chain (the oxy group was either substituted by a thioether or a secondary amino moiety) and in the pyridazinyl-piperidinyl moiety (left side in Figure 1). In some substances the methyl-isoxazol group present in the pleconaril molecule was added (Figure 1). Pirodavir (R 77975) [20] was kindly provided by the Janssen Research Foundation (Beerse, Belgium) and was used as a control in the various tests. The drugs were dissolved in DMSO at a concentration of 25 mg/mL and then stored in a refrigerator until use. 

### 3.2. Cells and viruses

The cytotoxic and antiviral activity of the compounds was performed on both the HeLa cells (Ohio strain) and the Hep-2 cells grown in DMEM with 1% non essential aminoacids, 200 μg/mL streptomycin, 200 units/mL penicillin G and 10% foetal calf serum (GIBCO Laboratories INC). Cell lines were kept at 37 °C in a humidified atmosphere with 5% CO_2_. The Rhinoviruses HRV14 (group A) and HRV39 (group B) and the Poliovirus strain Sabin 1, were purchased from the American Type Culture Collection (ATCC). For all the above mentioned viruses, working stocks were prepared as cellular lysates using DMEM with 2% heat inactivated fetal calf serum.

### 3.3. Cytotoxic activity

The cytotoxicity of the test compounds was evaluated by measuring the effect produced on cell morphology and cell growth in vitro. Cell monolayers were prepared in 24-well tissue culture plates and exposed to various concentrations of the compounds. Plates were checked by light microscopy after 24, 48 and 72 h. Cytotoxicity was scored as morphological alterations (e.g. rounding up, shrinking, detachment). The viability of the cells was determined by a tetrazolium-based colorimetric method using 3-(4,5-dimethylthiazol-2-yl)-2,5-diphenyltetrazolium bromide (MTT), as previously described [21,22]. The 50% cytotoxic dose (CD_50_) is the concentration of the compound that reduced the absorbance of the control sample by 50%. 

### 3.4. Inhibition of viral multiplication

The Rhinovirus inhibition assay was evaluated by a one-step viral infection of cell monolayers and then by virus yield titration in an agar-plaque assay. HeLa cell monolayers were prepared in 24 multiwell plates and were infected by the Rhinoviruses at a MOI of 1. Then serial dilutions of the test compounds were added and after 24–36 h of incubation at 33 °C and 3% CO_2_, when the cytopathic effect in the control cells was almost total, the monolayers were frozen and thawed and viruses in the supernatant were titrated by the plaque assay method. The assay of the antiviral activity of Poliovirus was carried out by the 50% plaque reduction assay in Hep2 cells as previously described [23]. The compound concentration required to inhibit virus plaque formation by 50% is expressed as the 50% inhibitory concentration (ID_50_) and calculated by dose-response curves and linear regression.

### 3.5. Time-reduction studies of virus yield

Time-reduction studies were performed on cell monolayers grown in 24 well plates. The compounds were added to the cells from time 0 to time +6 after viral infection. The cells were incubated in a CO_2_ atmosphere for 24–36 h, washed twice with HBSS and then the virus yield was detected by plaque assay.

## 4. Conclusions

This study confirms that the central chain of pirodavir and its derivatives seems to be the crucial part of these molecules for exerting a specific anti-Picornavirus activity. This behaviour can be due to the different structure of the “*canyon*” formed by the VP1 protein of the various species of Picornavirus strains [10]. Thus, considering that the VP1 viral canyons of different species of Picornaviruses have a peculiar structure in terms of size, depth and shape, we are designing novel SAR studies targeted to modify the central chain of new derivatives of compound I-6602, with the aim of finding original molecules that can better adapt inside these canyons, with a further possible improvement of their antiviral properties against the single Rhinovirus strains and hopefully also against other Picornavirus species. Finally, additional tests still have to be performed to define the stability and bio-availability of these newly synthesized compounds.

## Figures and Tables

**Table 1:** Screening of cytotoxic activity and anti-Rhinovirus properties of the novel compounds and the control Pirodavir.

Compounds	Cytotoxic activity	Anti-Rhinovirus activity				Anti-Poliovirus Activity	
	^a^CD_50_ μg/mL	HRV14 ^b^ID _50_ μg/mL	^c^PI	HRV39 ID_50_ μg/mL	PI	ID_50_ μg/mL	PI
I-6002	1.5	1.5	1	1.5	1	>1.5	<1
I-6230	6.0	6	1	3.1	2	0.5	12
I-6232	50.0	12.5	4	3.1	16	0.7	71
I-6273	50.0	3.1	16	1.5	32	1.4	35
I-6373	12.5	1.5	8	0.37	33	1.4	9
I-6473	6	1.5	4	0.75	8	1.4	4
I-6501	6	1.5	4	0.15	40	2.0	3
I-6502	12.5	0.15	83	0.07	17	2.6	4.8
I-6602	50	0.07	714	0.07	714	1.9	26
I-6702	50	0.07	714	0.35	142	9.1	5.5
PIRODAVIR	3.1	0.012	250	0.006	500	0.1	31

^a^ CD: Cytotoxic dose on HeLa cells; ^b^ ID: Inhibitory dose; ^c^ PI: Protection index (CD/ID).
